# Adaptation and validation of the Van Rie tuberculosis stigma scale in healthcare workers in Indonesia

**DOI:** 10.3389/fpubh.2025.1592191

**Published:** 2025-10-09

**Authors:** Siwi Pramatama Mars Wijayanti, Suratman, Dwi Sarwani Sri Rejeki, Budi Aji, Rosita Dwi Jayanti

**Affiliations:** ^1^Department of Public Health, Faculty of Health Sciences, Universitas Jenderal Soedirman, Purwokerto, Indonesia; ^2^Research Center of Rural Health, Jenderal Soedirman University, Purwokerto, Indonesia

**Keywords:** health workers, instrument, stigma, stigmatization, Indonesia, tuberculosis

## Abstract

**Objectives:**

Stigma related to tuberculosis (TB) is not limited to society and the workplace but also extends to healthcare settings. Stigma can result in delayed diagnoses, poor medication adherence, and a reduced quality of life. Currently, there is no instrument available to assess TB stigma among healthcare workers. This study aimed to adapt and validate a tuberculosis stigma scale specifically for health workers in Indonesia.

**Methods:**

This study used *Exploratory Sequential Mixed Methods.* Instrument development was carried out in three stages: translation, cross-cultural adaptation, and psychometric evaluation. The instrument adapted and validated in this study was the standardized Van Rie questionnaire. A total of 305 respondents from three areas such as Banyumas Regency, Yogyakarta City, and Malang City participated in this study. To assess the tool’s internal consistency and reliability, a psychometric evaluation was conducted using exploratory and confirmatory factor analyses (EFA and CFA).

**Results:**

Isolation and exclusion from medical facilities are the two categories of questions that have been identified. The results of the CFA demonstrated that the calculated chi-square value for our model was chi-square/DF = 186.713/43 = 4.3 (>3). The model was reasonably fit based on the following findings: the determining root mean square error of approximation (RMSEA) = 0.105 (>0.08), normed fit index (NFI) = 0.837 (<0.90), tucker-lewis index (TLI) = 0.832 (<0.95), and standardized root mean square residual (SRMR) = 0.080 (<0.10). The instrument was reliable with a Cronbach’s alpha of 0.829.

**Conclusion:**

This validated, consistent, and reliable adapted tool is ready to use in larger-scale evaluation of TB-related stigma among health workers in healthcare settings to develop strategies to eliminate TB-related stigma.

## Introduction

The issue of tuberculosis (TB) in Indonesia remains a national priority, necessitating collaborative efforts from various stakeholders to address it. Indonesia has set a target to eliminate TB by 2030 (1). Although the global prevalence of tuberculosis is decreasing, it remains high in Indonesia, despite increased funding, improved healthcare access, enhanced monitoring, better diagnosis, situational analysis, and focused policies. Several challenges persist, including gaps in the identification and treatment of tuberculosis: only 34% of patients receive successful treatment, and 28% of cases remain undiagnosed (2, 3). Furthermore, incomplete treatment regimens, unintegrated referral programs, undetected multidrug-resistant cases, insufficient treatment for patients with HIV and tuberculosis, and low uptake of preventive treatment all contribute to the limited availability of high-quality tuberculosis treatment coverage (1). Directly observed therapy (DOTS) is the method used to manage tuberculosis (TB) in endemic areas. Indonesian government commitment, case detection, standardized short-course chemotherapy, and a monitoring system for program oversight and evaluation are important elements of this strategy (4).

The World Health Organization (WHO) and the United Nations have identified TB stigma as a major obstacle to the global eradication of tuberculosis (5). The stigma attached to having this illness is one of the hypothesized reasons why people put off getting treatment. Since TB stigma is such an ethereal term, it is challenging to define. The World Health Organization explains that stigma is a form of social judgment—something that makes people feel ashamed, rejected, or looked down upon. For those affected by tuberculosis, stigma can have serious consequences. It not only harms their health and wellbeing but also makes it harder to fight the disease overall. Many people fear losing their jobs, relationships, homes, or even access to education if others find out they have TB. As a result, they are often too afraid to get tested or seek treatment, which only makes the illness harder to manage (6). Regarding tuberculosis (TB), stigma arises from the widely held belief that the disease carries a “death penalty” and is associated with unhealthy habits and poor people, making it a “dirty disease.” There have been reports that even after TB is healed, the stigma associated with the illness may endure (7).

Stigma in society can occur in the general public, in the work environment, or even in health workers and health services. Several previous studies have mentioned the impact of TB stigma on disease detection, regularity in taking medication, and treatment success (8–10). Stigma can also reduce the quality of life of TB sufferers (11). Measuring stigma is essential to comprehending its causes and prevalence and evaluating the success of measures aimed at reducing it. There are several instruments and scales available to measure stigma connected to health (12). These tools need to be developed to make sure they are accurate, specific, and dependable before being verified in the community or population in which they are to be utilized. Only then could they be considered robust and reliable. Various instruments and scales have been created to evaluate tuberculosis stigma. Nonetheless, in order to guarantee their precision, dependability, and resilience, these scales and instruments must be modified, verified, tested, and improved before being expanded within a particular community or demographic. Van Rie’s TB Stigma Scale is one of the widely used instruments. This scale has been validated in several languages and contexts, including Thailand, Portugal, Mexico, Turkey, and Vietnam, and it has demonstrated strong internal consistency (13, 14).

In Indonesia, validation of the Van Rie questionnaire has been carried out in the community and in the work environment (12, 15), but no one has carried out the development of a tool to measure and assess TB stigma in the healthcare setting. Validating and adapting TB stigma measures for healthcare settings is essential, as the nature of stigma in these environments differs from that in the general population or typical workplaces. While some aspects may overlap with workplace stigma, healthcare workers—especially those who interact directly with TB patients—face unique challenges. The way they experience and respond to stigma can deeply influence how TB care is provided and how patients are treated within the system. Since TB management necessitates close collaboration between patients and healthcare workers (HCWs), assessing TB stigma among health workers is crucial. There could be detrimental effects from the stigma among HCWs (16). Stigma at healthcare facilities takes many different forms, some of which are well-documented and include outright denial of care, subpar care, verbal and physical abuse, and more subtle forms such as making some patients wait longer or delegating their care to less experienced staff members (17). Therefore, it is necessary to validate the tuberculosis stigma questionnaire among health workers, measure TB stigma among health workers, and try to implement activities to reduce stigma. Building on the background presented, this study aims to develop a measurement instrument to assess tuberculosis-related stigma among healthcare workers in Indonesia through a process of adaptation and validation. The development process includes translation, cross-cultural adaptation, and psychometric evaluation. Developing a valid questionnaire for important specific population health workers is expected to be able to more accurately capture the true stigma that occurs among HCWs. It is anticipated that this ready-to-use instrument will serve as a valuable tool in the prevention and management of tuberculosis-related stigma, particularly among healthcare workers.

## Materials and methods

### Design and location of the study

**Figure 1:**
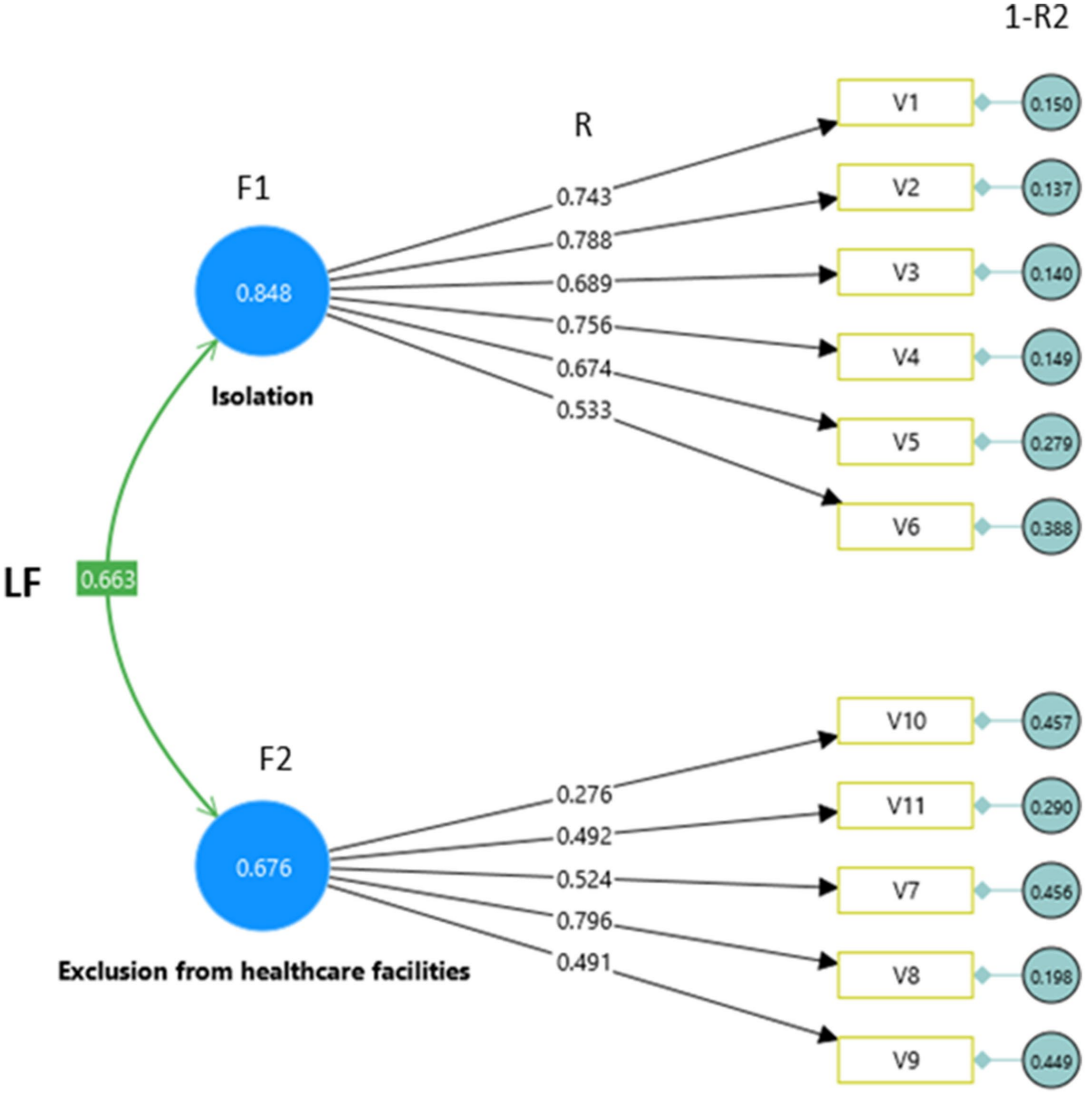
Confirmatory factor analysis of the tool. LF: covariance between factors; F: loading factors; V: tool’s item; R: variance indicating magnitude of relationship of items to factor; 1-R2: percentage of variance of each item not explained by factor.

This study used an exploratory sequential mixed methods design, beginning with a qualitative phase that involved translating the instrument and gathering feedback from experts. This was followed by a quantitative phase, where the instrument was pilot-tested and underwent psychometric evaluation. This study was conducted in three areas: Banyumas Regency in Central Java, Yogyakarta City in the Special Region of Yogyakarta, and Malang City in East Java. The three phases of this study—translation, cross-cultural adaptation, and psychometric evaluation—were carried out between March and October of 2024. Phase 1 was conducted over 3 weeks, followed by Phase 2 for 1.5 months and Phase 3 for 3 months. Figure 1 illustrates the location of the research site.

### Instrument development

**Figure 2:**
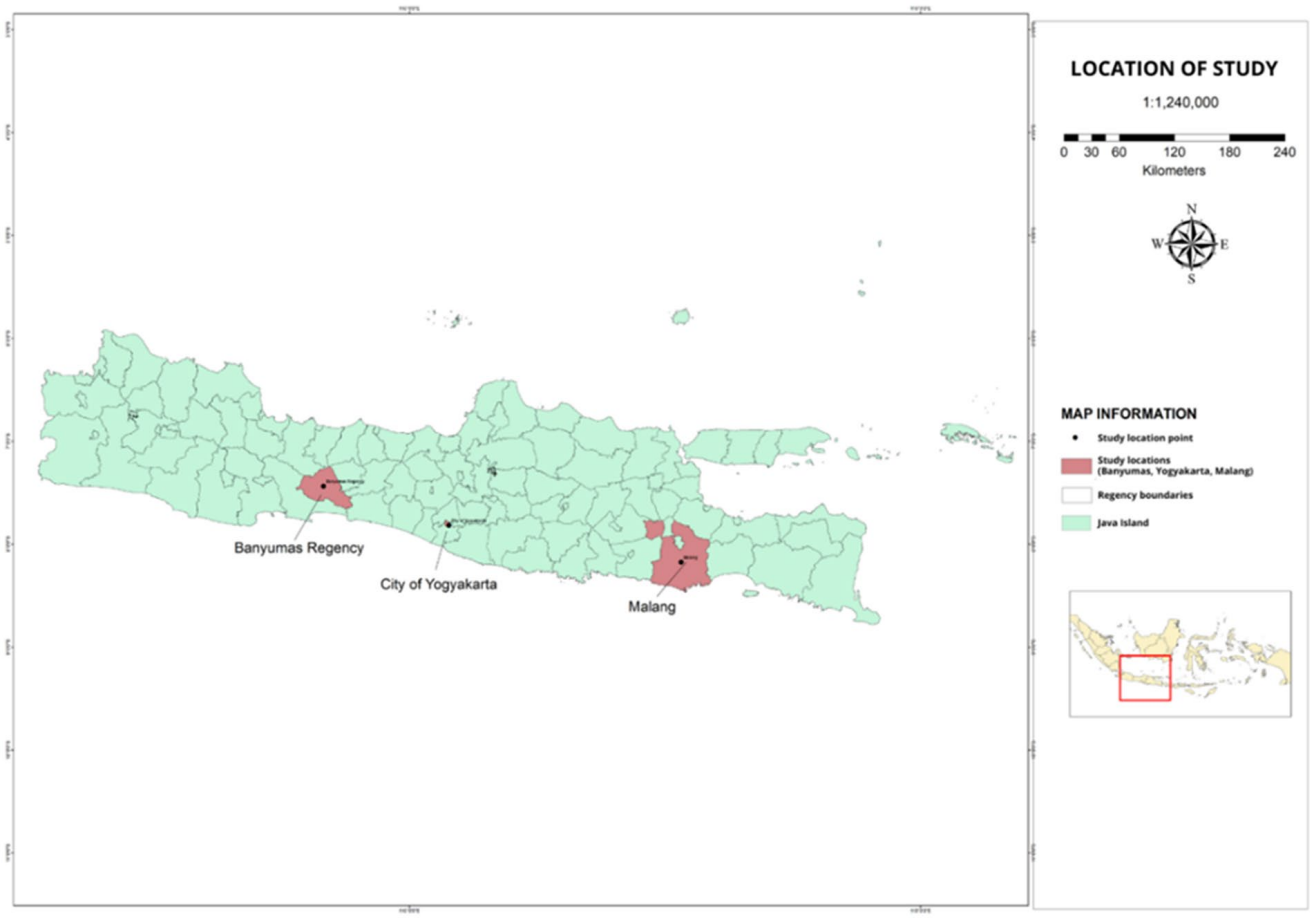
The locations where the psychometric evaluation was carried out were in Banyumas Regency, Yogyakarta City and Malang City. The red area is the location where the psychometric test of the developed instrument was carried out.

We adapted Van Rie’s Stigma Scale, which originally consisted of two parts: Part A: community perspectives toward TB (11 items) and Part B: patient perspectives toward TB (12 items). We used Part A for adaptation and validation in the health worker. Each of the 11 items in Part A of the Van Rie Stigma Scale has four options: strongly disagree (0), disagree (1), agree (2), and strongly agree (3). The stages of instrument development are shown in Figure 2.

#### Phase 1—Translation

The instrument in Part A (community perspective toward TB) was translated into Bahasa, Indonesia’s official language. This stage was carried out by two independent researchers with extensive research experience and competent English, resulting in two versions of the translation results. Then, an internal discussion was held by the research team to discuss the two translation results. Based on the outcomes of the discussion, the research team came to a consensus regarding the statement that would be included in the instrument that was created. The discussion also included the context of the specific population—health workers—that would be used in this study. The research team produced one version of the draft instrument that was compiled based on the results of the agreement. Then, back translation into English was carried out by a sworn translator.

#### Phase 2—Cross-cultural adaptation

We adapted the formulated instrument for the Indonesian context by inviting several experts to provide input and suggestions. In the expert meeting, we invited health service staff from the infectious disease control division specifically dealing with tuberculosis and several representatives of the heads of community health centers. The research team presented the draft instrument that had been developed and then asked for input from all invitees. After considering the experts’ feedback, the research team revised the instrument and conducted internal consolidation to create the final testing version. Expert input in the form of language improvements makes it easier to understand by replacing some terms and adjusting the context of stigma in health facilities. There were two additional questions at the final stage of finalizing the instrument:

*10. I assume that patients suffering from TB are at risk of transmitting the disease to others in health services.*

*11. I assume that the presence of patients suffering from TB in the Health Center leads to fear among the community members in receiving health services.*

#### Phase 3—psychometric evaluation

##### Respondent selection and sample size

A sample calculation was carried out using the formula for estimating a population proportion with specified absolute precision. The minimum sample size was calculated using a 95% confidence interval level; the proportion of health workers who have stigma-related tuberculosis was 0.5; and the degree of precision was 10%. Based on the result of the minimum sample calculation, the minimum number of samples for this study was 96 respondents, which we rounded up to 100 respondents for each area. Inclusion criteria of this study are health workers employed at health centers and directly involved in patient care (including doctors, nurses, midwives, and other relevant health center staff). Eligible participants must be aged 18 years or older and must provide informed consent to voluntarily participate in the research. Administrative staff at health centers who do not serve patients, such as financial managers and general affairs staff, were not included in this study. We also gathered information on respondent characteristics including gender, age, education, job section at the health center, sources of information obtained regarding tuberculosis, and family history or whether or not someone close to them had TB.

### Data collection and statistical analyses

Field enumerators—ranging from six to eight per area—assisted with data collection. To ensure they fully understood the research procedures and data collection techniques, the research team provided training prior to the start of fieldwork. Informed consent contains research information for respondents and assurance of respondents’ willingness to participate before data collection. Permission has also been obtained from the health service and the community health center where the research was conducted.

The internal consistency of the instrument was measured by performing exploratory factor analysis (EFA). A threshold for Kaiser–Meyer–Olkin’s (KMO) and Bartlett’s test values was set at 0.7 and 0.05, respectively, in the principal axis factor analysis. To determine the number of factors, we assessed the eigenvalues. Factors with eigenvalues of more than one and containing a loading of more than or equal to 0.4 were included.

Furthermore, a confirmatory factor analysis (CFA) was used to evaluate a model by determining root mean square error of approximation (RMSEA) < 0.05: an excellent fit, RSMEA = 0.05–0.08: an acceptable fit, RMSEA = 0.08–0.1: a marginal fit, and RMSEA > 0.1: a poor fit. A test of reliability used Cronbach’s alpha, with a Cronbach’s alpha coefficient of 0.80–0.90 being considered reliable. CFA was performed using SmartPLS 4.

### Ethical considerations

This study has received ethical approval from the Ethics Committee of the Indonesian National Research and Innovation Agency Number (No 105/KE.03/SK/05/2024). To respect the freedom of respondent participation, we provide information related to participation in the study in the informed consent, and respondents who are willing to participate will sign a willingness sheet. To maintain the confidentiality of respondents, we do not include the identity of respondents in the preparation of research reports or in publications.

## Results

The development of the TB stigma measurement instrument for health workers has been carried out in three stages: translation, cross-cultural adaptation, and psychometric evaluation. In the psychometric evaluation stage, researchers tested the developed instrument on 305 respondents whose characteristics are shown in Table 1.

**Table 1:** Characteristic of respondents (*n* = 305).

Characteristics	Number (*n*)	Percentage (%)
Sex		
Male	51	16.7
Female	254	83.3
Education level		
Senior high school	2	0.7
Diploma III	83	27.2
Diploma IV	2	0.7
Undergraduate	214	70.2
Postgraduate	4	1.3
Length of employment (year)		
1–5	110	36.1
6–10	64	21.0
>10	131	43.0
Types of occupations		
Doctor	59	19.3
Nurse	115	37.7
Midwife	36	11.8
Other health personnel	95	31.1
Directly serving TB patients		
Yes	170	55.7
No	135	44.3
Family members or close relatives were previously diagnosed with TB		
Yes	35	11.5
No	270	88.5
Sources of Information about TB		
Colleagues	263	86.2
Website/internet	232	76.1
Social media	208	68.2
Poster/flyer	197	64.6
Television (TV)/radio	76	24.9

Most of the respondents interviewed were female (83%), had a bachelor’s degree (70%), and had worked for more than 10 years (43%). A total of 37.7% of respondents worked as nurses in the community health center, 55.7% directly served TB patients in their work, 11.5% had family/relatives who previously had TB, and 86.2% stated that they received information about TB from their colleagues at work.

We measured the internal consistency of the instrument by performing EFA. EFA for the 11-item adapted stigma instrument demonstrated a KMO value of 0.862 and a Bartlett’s test value of 1128.026 (*p <* 0.001). Furthermore, two loading factors were isolation (V1, 2, 3, 4, 5, and 6) and exclusion from healthcare facilities (V7, 8, 9, 10, and 11) (Table 2). The instrument was reliable with a Cronbach’s alpha of 0.829. The results of the CFA demonstrated that the calculated chi-square value for our model was chi-square/DF = 186.713/43 = 4.3 (>3). The model was reasonably fit based on the following findings: the RMSEA = 0.105 (>0.08), NFI = 0.837 (<0.90), TLI = 0.832 (<0.95), and SRMR = 0.080 (<0.10). The loading factors of the 11 question items are shown in Table 2.

**Table 2:** Loading factors of each questions instrument.

		Component		Cronbach’s alpha if item deleted
		1	2	
V1	I refuse to provide services to patients suffering from Tuberculosis (TB)	0.814		0.805
V2	I try to stay away from patients suffering from TB	0.819		0.797
V3	I feel that patients suffering from TB are embarrassed	0.780		0.808
V4	I choose to serve other patients than those suffering from TB	0.782		0.797
V5	I reduce direct interaction (talking/communicating) with patients suffering from TB	0.657		0.793
V6	I treat patients suffering from TB differently	0.506		0.803
V7	I refuse to eat and drink with patients suffering from TB		0.709	0.815
V8	I avoid direct physical contact (touching) with patients suffering from TB	0.529	0.529	0.791
V9	I am afraid of being infected by patients suffering from TB		0.671	0.816
V10	I assume that patients suffering from TB are at risk of transmitting the disease to others in health services		0.711	0.834
V11	I assume that the presence of patients suffering from TB in the Health Center leads to fear among the community members in receiving health services	0.342	0.461	0.811

### Goodness of fit untuk CFA

**Figure 3:**
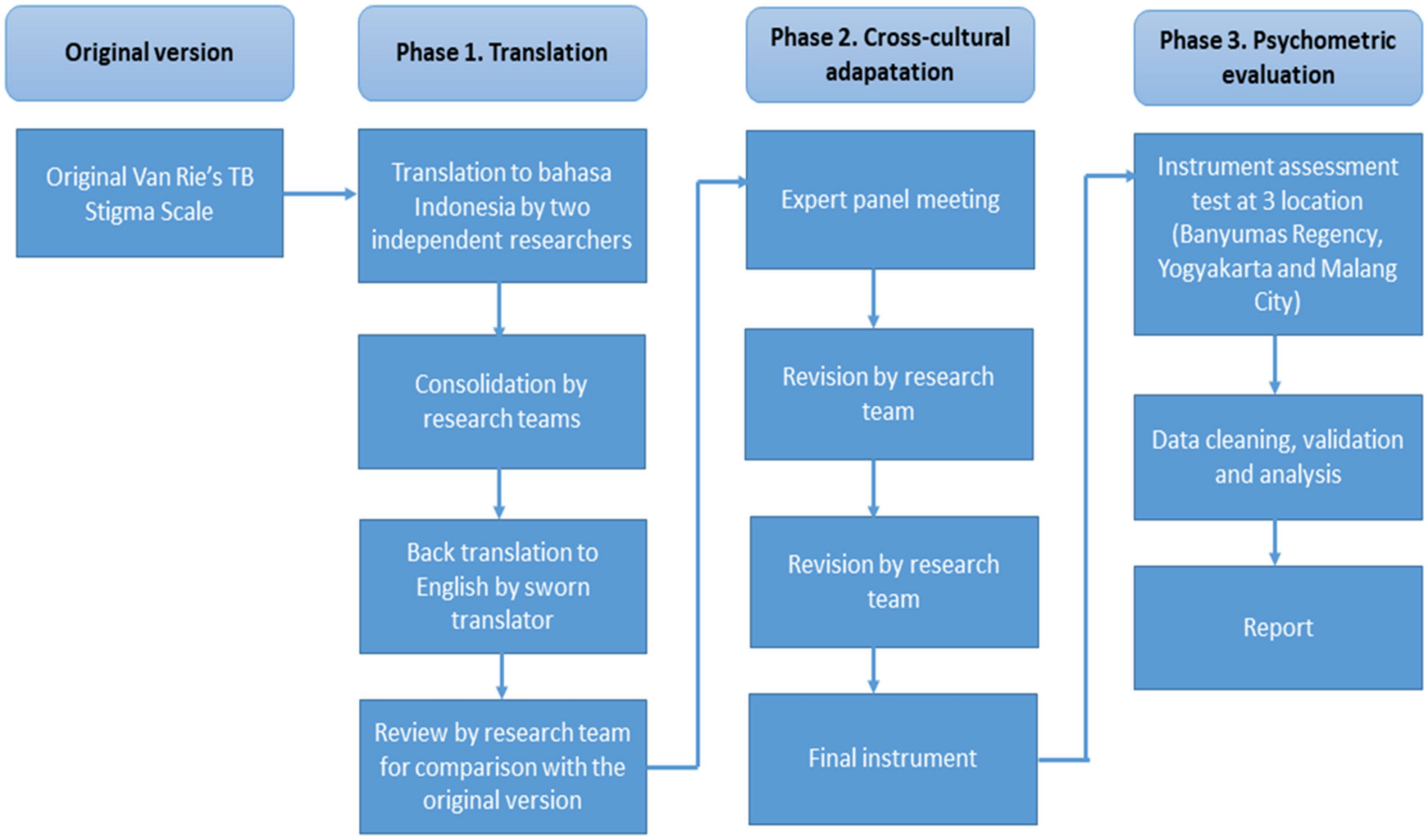
Instrument development flow in this study.

The results obtained are RMSEA = 0.105 (>0.08), NFI = 0.837 (<0.90), TLI = 0.832 (<0.95), SRMR = 0.080 (<0.10), and chi-square/DF = 186.713/43 = 4.3 (>3). In general, the resulting model is still reasonably fit (Figure 3). Based on several instrument analyses that have been carried out, the questionnaire produced is valid, has good internal consistency, and is reliable.

## Discussion

Based on the study conducted, the Van Rie Tuberculosis (TB) Stigma Scale was successfully adapted and validated for healthcare workers in Indonesia, demonstrating strong internal consistency and reliability. The newly developed tool is capable of assessing TB-related stigma specifically within healthcare settings, providing valuable insights to help reduce stigma and improve the quality of care and treatment adherence among TB patients in Indonesia. We developed a new tool for Indonesian health professionals by culturally adapting Van Rie’s TB Stigma Scale. The new tool featured certain modified and extra elements relevant to health workers, was deemed comprehensive, and had strong internal consistency and content validity. This is the first TB stigma measurement tool used in Indonesian healthcare settings. The Van Rie TB stigma scale instrument has been modified in two investigations conducted in Indonesia: Fuady et al. modified the instrument for TB stigma in general (15), and Soemarko et al. modified it for use in the workplace (12). This indicates that although stigma still occurs frequently in Indonesia, adaptation of stigma instruments and measurement is still very limited.

Adaptation and validation of the new instrument in the new context were carried out by referring to the ISPOR (International Society for Pharmacoeconomics and Outcomes Research) guidelines. Following ISPOR guidelines ensures that the adapted tool is not only scientifically valid but also culturally relevant and practically applicable in the new context. Adaptation is necessary when a measurement tool is used in a new population or setting that differs from the original context where it was developed. This ensures that the tool is culturally and contextually relevant. The adaptations made in the instruments developed were to adapt them to the context of healthcare settings. We also refer to the process of instrument adaptation and validation in several studies that did similar things (18–20).

We adapted the context of the healthcare setting by deciding to ask respondents in the first person “I,” changing from Van Rie’s original questionnaire, which used “some people.” Asking questions to respondents using the subject “I” as the first person aims to directly capture their personal perceptions when dealing with and serving TB patients while carrying out their daily tasks. We also added two questions to adapt to the context of the healthcare setting: “I believe that patients suffering from Tuberculosis are at risk of transmitting the disease to other patients in health services” and “I believe that the presence of TB patients at the Community Health Center may make it can cause people to be afraid to go to health services.” This was also done by Soemarko et al. when adapting the instrument to a work environment population (12). We also received several inputs for instrument development from many experts in the field of TB. In the expert meeting, we identified cultural factors, language differences, and contextual variables that may influence the tool’s interpretation and application. We involved stakeholders (TB staff in health offices, TB staff in community health centers, and researchers) to gather insights on the instrument’s relevance and potential modifications. These steps follow the recommendations for the development stages of a new instrument (21).

The EFA showed two main factors running throughout the 11 items: isolation and exclusion from healthcare facilities. These two key elements are nearly identical and might be connected. The factors of isolation and exclusion from the healthcare facilities are useful for identifying the roots of stigma in healthcare settings. Isolation factors include refusal to provide health services, avoiding, considering having TB as something shameful, and treating them differently from other patients. This is possible due to the fear of being infected by TB patients as well as misinformation regarding the transmission and risks of TB (5). The previous research showed that the lack of that knowledge leads to fear and stigma. The reported enacted (isolation and gossip) and expected (concealment of treatment and self-isolation) stigmas were caused by fear of contracting tuberculosis (22). People who do not understand how TB is transmitted or treated may stigmatize those with TB. Even people who understand how TB is transmitted may still stigmatize people with TB if they perceive a high risk of transmission. Health workers should have good knowledge about TB transmission, but fear of infection can still create stigma in TB patients.

TB stigma can exist in healthcare settings as well as in society and the workplace; this tool will be very helpful in evaluating TB that may arise in these situations. The reason for the emergence of stigma among healthcare workers toward TB patients, based on several studies, is the fear of being infected while providing services (23, 24). In the healthcare setting, TB-related stigma is frequently linked to “dirty work,” and healthcare workers who provide TB care are perceived as having to deal with this stigma (23). TB stigma in healthcare settings can result in treatment non-adherence and delayed diagnosis. Additionally, it might make it more difficult for patients to manage their illness by causing financial hardships and destroying social ties (25). Patients with stigmatized tuberculosis are unwilling to seek and finish therapy (8, 26). This study highlights how TB stigma among health workers is shaped by unique workplace factors, including high workloads, institutional pressures, organizational dynamics, and interpersonal relationships. Unlike stigma in the general population, health workers’ attitudes toward TB are influenced by their professional responsibilities, fear of infection, and systemic demands that may prioritize efficiency over empathy (23). Institutional culture and peer dynamics can either reinforce or reduce stigma, depending on the level of support, communication, and leadership. These findings suggest that effective stigma reduction in healthcare settings requires not only individual-level interventions but also structural changes that address the broader organizational environment in which health workers operate (27).

We successfully adapted Van Rie’s TB Stigma Scale into a new tool to measure TB stigma among health workers in healthcare settings in Indonesia. This instrument is ready to be used to measure TB-related stigma among health workers in Indonesia. It could be necessary to test the tool outside of Java to determine its viability and adjust it to the local environment. In conclusion, the adaptation and validation of the Van Rie Tuberculosis Stigma Scale for healthcare workers in Indonesia have resulted in a reliable and culturally appropriate tool. This instrument is now ready for use in large-scale evaluations to assess and address TB-related stigma within healthcare settings, ultimately contributing to more effective TB management and care.

## Data Availability

The raw data supporting the conclusions of this article will be made available by the authors, without undue reservation.
